# Systemic immune inflammation index and system inflammation response index on the third postoperative day predict poor prognosis of aneurysmal subarachnoid hemorrhage patients

**DOI:** 10.1097/MD.0000000000037818

**Published:** 2024-04-19

**Authors:** Xian Wang, Wei Tian, Yongfeng Zhao, Yong Yang, Li Deng

**Affiliations:** a Department of Pharmacy, The First Affiliated Hospital of Yangtze University, Jingzhou, China; b The Neurointensive Care Unit, The First Affiliated Hospital of Yangtze University, Jingzhou, China; c Department of Hematology, The First Affiliated Hospital of Yangtze University, Jingzhou, China; d Medical Department, The First Affiliated Hospital of Yangtze University, Jingzhou, China.

**Keywords:** aneurysmal subarachnoid hemorrhage, neutrophil-lymphocyte ratio, platelet-lymphocyte ratio, system inflammation response index, systemic immune inflammation index

## Abstract

The inflammatory response is involved in the progression of aneurysmal subarachnoid hemorrhage (aSAH). We sought to investigate the relationships of inflammatory indicators including blood cell counts and the ratios of different blood cells counts with the prognosis of aSAH patients. We performed a retrospective study including 140 patients with aSAH and aneurysm surgeries. The relationships of neutrophils, lymphocytes, monocytes, platelets, systemic immune inflammation index (SII), system inflammation response index (SIRI), neutrophil-lymphocyte ratio and platelet-lymphocyte ratio with prognosis were investigated by univariable analysis and multivariable logistic regression model. The patient with Modified Rankin Scale (mRS) score＜3 was defined as having a good prognosis, while with mRS score ≥3 was defined as having a poor prognosis. Among 140 patients included, there were 108 cases with good prognosis and 32 cases with poor prognosis after follow-up. On the 3rd postoperative day, the neutrophils counts, SIRI level and SII level in cases with poor prognosis were significantly higher than cases with good prognosis, *P* < .05. After adjusting for baseline differences in Hunt-Hess grade, Glasgow Coma Scale score, combination with intraventricular hemorrhage and maximum diameter of aneurysm, the levels of SIRI (odds ratio = 3.968, 95% CI: 1.432–10.992, *P* = .008) and SII (odds ratio = 3.313, 95% CI: 1.029–10.665, *P* = .045) on the 3rd postoperative day could predict poor prognosis. SII and SIRI on the 3rd postoperative day could independently predict the poor prognosis in aSAH. However, the cutoff values for predicting prognosis needs to be validated in larger-sample studies.

## 1. Introduction

Intracranial aneurysm is aneurysmal protrusion formed by localized pathological dilation of the intracranial arterial wall. Aneurysmal subarachnoid hemorrhage (aSAH) is caused by rupture of the aneurysm and accounts for approximately 80% of subarachnoid hemorrhage (SAH).^[1]^ The worldwide annual incidence of aSAH was (2–16)/100,000, accounting for 8% of all stroke.^[2]^ SAH could account for 2% of stroke and tended to occur in patients over 50 years old in 1 community study in 0.5 million Chinese adults.^[3]^ The mortality of aSAH was 34% in a systematic review including 85 clinical studies and 4506 patients with poor-grade aSAH.^[4]^

Inflammatory response was involved in the progression of SAH and possibly associated with prognosis of patients.^[5]^ One study carried by Dhar R et al showed that systemic inflammatory response syndrome occurred in more than half of SAH patients, 85% of which happened within the first 4 days. All patients with vasospasm had evidence of systemic inflammatory response syndrome and delayed ischemic neurological deficit.^[6]^ Moreover, the results of another study showed that systemic inflammatory response syndrome was associated with poor Hunt-Hess grade, Fisher grade, higher mortality of patients and increased risk of cerebral vasospasm, hydrocephalus and systemic complications.^[7]^ Neutrophils, lymphocytes and neutrophil-lymphocyte ratio were possibly involved in the neuroinflammatory response after cerebral hemorrhage and were associated with prognosis of patients.^[8,9]^ The inflammatory response varied at different times of hemorrhage. There were limited studies on the relationships of these inflammatory indicators with prognosis of aSAH patients during different times. In our study, we investigated the relationships of blood cell counts and other inflammatory indicators before and after surgeries with the prognosis of aSAH patients.

## 2. Materials and methods

### 2.1. Study design and participants

**Figure 1.**
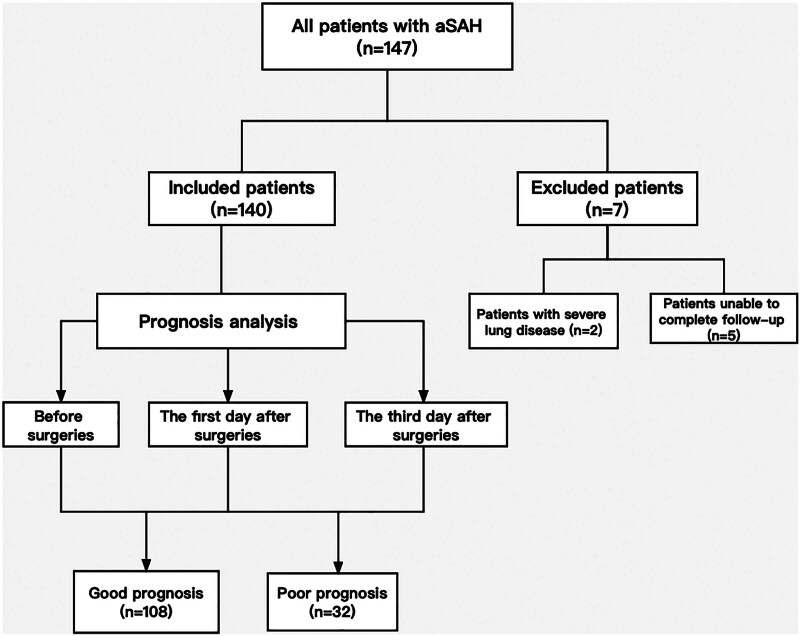
Flow diagram of the study.

One retrospective study including 140 aSAH patients with surgeries in the Neurointensive Care Unit of the First Affiliated Hospital of Yangtze University from 2020 to 2022 was carried. The inclusion criteria of patients were: ≥18 years old; admitted within 72 hours of the onset of symptoms; had subarachnoid hemorrhage caused by aneurysm rupture confirmed by Digital Subtraction Angiography and/or Computed Tomographic Angiography examinations; received clipping or embolization surgeries after admission. Exclusion criteria of patients were: had subarachnoid hemorrhage caused by trauma or perimesencephalic nonaneurysmal subarachnoid hemorrhage; suffered from severe systemic diseases before hospital admission, such as severe liver or kidney dysfunction, heart dysfunction, lung diseases, etc; unable to complete follow-up. The prognosis was determined by the Modified Rankin Scale (mRS) results from 90-day telephone or outpatient follow-up. The patients with mRS score of 0 to 2 were determined to have a good prognosis, and patients with mRS score of 3 to 6 were determined to have a poor prognosis. The mRS score was based on the following condition: asymptomatic (score: 0); with the ability to complete all frequently engaged activities (score:1); mildly disabled and unable to complete all activities that have been previously engaged in, but able to handle personal affairs without need for assistance (score: 2); moderately disabled and required some assistance, but can walk without assistance (score: 3); severely disabled and unable to walk without assistance, with inability to take care of own physical needs (score: 4); severely disabled, bedridden, incontinence, and with the need for continuous care and attention (score: 5); death state (score: 6).^[10]^ The flow chart of patients was showed in Figure 1.

### 2.2. Data collection

Baseline characteristics included age, gender, complications (hypertension, diabetes, hyperlipidemia), aneurysm parameters (maximum diameter and neck length), time length of surgery, Hunt-Hess grade, score of Glasgow Coma Scale (GCS) and combination with intraventricular hemorrhage. Blood cells counts from Automatic Blood Cell Analyzer (SYSMEX XN-9000) were extracted from the hospital information system, including neutrophils, lymphocytes, monocytes and platelets on the preoperative day, the 1st and 3rd postoperative days. The neutrophil-lymphocyte ratio (NLR) and platelet-lymphocyte ratio (PLR) were calculated. Systemic immune inflammation index (SII) and system inflammation response index (SIRI) were also calculated. SII = platelet count × NLR. SIRI = monocyte count × NLR. The prognosis was determined by the Modified Rankin Scale (mRS) results of 90-days follow-up. The patient with mRS (0–2) was defined as having a good prognosis, while with mRS (3–6) was defined as having a poor prognosis. There were a total of 108 patients with good prognosis and 32 patients with poor prognosis.

### 2.3. Statistical analysis

SPSS 23.0 (IBM SPSS Inc., Chicago, USA) was used for data analysis. If the quantitative variables met the normal distribution and variances were homogeneous, independent-sample t-test was used for analysis. If the quantitative variables met the normal distribution but variances were not homogeneous, corrected *t* test was used. If the normal distribution could not be satisfied, Mann–Whitney *U* test was used for analysis. The qualitative variables were compared by Pearson chi-square test, Continuity correction or Fisher exact test. The multivariate logistic regression model was constructed for factors with poor prognosis. Covariates that had a *P*-value <.05 in the univariate analysis were added to the multivariate logistic analysis. Individual multivariable model was built for each inflammatory indicator keeping all other factors the same. Receiver operating characteristic (ROC) was performed to assess the ability of factors with significance to distinguish patients with poor prognosis from good prognosis. All *P*-values were 2-sided and the statistical significance was set at *P* < .05. Youden’s index was calculated to determine optimal test cutoff value.

## 3. Results

### 3.1. Study participants and characteristics

As showed in Table 1, there were 53 males and 87 females among included patients. The average age was 59 years. Among these patients, there were 93 cases with Hunt-Hess grade I–II and 47 cases with Hunt-Hess grade III–V, 117 cases with GCS score ≥11 and 23 cases with GCS score < 11. There were 68 cases combined with intraventricular hemorrhage. The median time length of surgery was 180 minutes. The preoperative counts of neutrophils, lymphocytes, monocytes and platelets were (10.71 ± 4.54) × 10^9^/L, (0.87 ± 0.55) × 10^9^/L, (0.45 ± 0.39) × 10^9^/L and (200 ± 69) × 10^9^/L respectively. On the 1st postoperative day, the counts were (8.89 ± 4.25) × 10^9^/L, (0.92 ± 0.39) × 10^9^/L, (0.66 ± 0.41) × 10^9^/L and (174 ± 69) × 10^9^/L respectively. On the 3rd postoperative day, the counts were (7.55 ± 3.81) × 10^9^/L, (1.03 ± 0.68) × 10^9^/L, (0.70 ± 0.26) × 10^9^/L and (176 ± 57) × 10^9^/L respectively. The other inflammatory indicators calculated from blood cell counts were showed in Table 2.

**Table 1:** Baseline clinical characteristics according to the prognosis of aSAH patients.

	All patients (n = 140)	Good prognosis (n = 108)	Poor prognosis (n = 32)	*P*-value
Gender (n [%])				
Male	53 (37.9)	38 (35.2)	17 (53.1)	.231
Female	87 (62.1)	70 (64.8)	15 (46.9)	
Age (yr)	59 ± 9	59 ± 10	61 ± 7	.353
Hunt-hess grade (n [%])				
III–V	47 (33.6)	23 (21.3)	24 (75.0)	<.001
I–II	93 (66.4)	85 (78.7)	8 (25.0)	
GCS score (n [%])				
≥11	117 (83.6)	99 (91.7)	18 (56.3)	<.001
＜11	23 (16.4)	9 (8.3)	14 (43.8)	
Combination with intraventricular hemorrhage (n [%])				
Yes	68 (48.6)	43 (39.8)	25 (78.1)	<.001
No	72 (51.4)	65 (60.2)	7 (21.9)	
Complications (n [%])				
Hypertension	62 (44.3)	43 (39.8)	19 (59.4)	.443
Diabetes	2 (1.4)	2 (1.9)	0 (0)	
Hyperlipidemia	8 (5.7)	7 (6.5)	1 (3.1)	
Two or more above diseases	15 (10.7)	13 (12.0)	2 (6.3)	
None	53 (37.9)	43 (39.8)	10 (31.3)	
Neck length of aneurysm	3.50 ± 1.63	3.50 ± 1.37	3.57 ± 2.87	.277
Maximum diameter of aneurysm (mm)	5.20 ± 3.24	4.69 ± 2.60	6.24 ± 2.89	.007
Time length of surgery (min)	180 ± 90	169 ± 78	190 ± 90	.122

**Table 2:** Associations of inflammatory indicators from blood cells counts with the prognosis of aSAH patients.

	All patients (n = 140)	Good prognosis (n = 108)	Poor prognosis (n = 32)	*P*-value
Before surgeries				
Neutrophils (×10^9^/L)	10.71 ± 4.54	10.09 ± 4.07	12.83 ± 5.39	.011
Lymphocytes (×10^9^/L)	0.87 ± 0.55	0.86 ± 0.54	0.98 ± 0.93	.316
Monocytes (×10^9^/L)	0.45 ± 0.39	0.42 ± 0.36	0.54 ± 0.49	.021
Platelets (×10^9^/L)	200 ± 69	202 ± 68	196 ± 77	.416
SIRI (×10^9^/L)	4.43 ± 6.65	4.14 ± 5.72	6.64 ± 10.46	.046
SII (×10^9^/L)	2215.19 ± 2254.51	2176.70 ± 2226.92	2415.05 ± 2640.87	.342
NLR	11.12 ± 9.52	10.86 ± 9.52	11.45 ± 9.96	.635
PLR	222.99 ± 163.50	229.20 ± 162.09	193.35 ± 172.34	.503
On the 1st day after surgeries				
Neutrophils (×10^9^/L)	8.89 ± 4.25	8.74 ± 3.65	10.77 ± 5.21	.005
Lymphocytes (×10^9^/L)	0.92 ± 0.39	0.94 ± 0.39	0.85 ± 0.38	.282
Monocytes (×10^9^/L)	0.66 ± 0.41	0.65 ± 0.38	0.70 ± 0.54	.147
Platelets (×10^9^/L)	174 ± 69	177 ± 66	161 ± 81	.935
SIRI (×10^9^/L)	7.06 ± 8.42	6.40 ± 6.60	10.49 ± 9.62	.006
SII (×10^9^/L)	1839.12 ± 1571.21	1782.43 ± 1473.77	2311.72 ± 2273.49	.019
NLR	10.44 ± 7.60	9.97 ± 6.46	13.10 ± 11.52	.009
PLR	196.54 ± 137.93	196.54 ± 116.58	199.69 ± 213.40	.490
On the 3rd day after surgeries				
Neutrophils (×10^9^/L)	7.55 ± 3.81	7.54 ± 2.85	9.78 ± 3.21	<.001
Lymphocytes (×10^9^/L)	1.03 ± 0.68	1.04 ± 0.68	0.86 ± 0.71	.039
Monocytes (×10^9^/L)	0.70 ± 0.26	0.67 ± 0.24	0.77 ± 0.31	.077
Platelets (×10^9^/L)	176 ± 57	178 ± 62	153 ± 92	.459
SIRI (×10^9^/L)	5.05 ± 5.11	4.44 ± 4.13	8.39 ± 6.75	<.001
SII (×10^9^/L)	1261.53 ± 974.95	1183.47 ± 866.79	1535.94 ± 2017.53	.002
NLR	7.56 ± 5.60	6.48 ± 5.14	10.23 ± 7.61	<.001
PLR	170.33 ± 101.63	166.35 ± 87.54	186.64 ± 192.62	.165

### 3.2. Relationships of inflammatory indicators and prognosis of aSAH patients by univariable analysis

There were no significant differences between patients with good and poor prognosis in gender (*P* = .231), age (*P* = .353), neck length of aneurysm (*P* = .277), and time length of surgery (*P* = .122). The proportions of Hunt-Hess grade III–V cases (*P* < .001), cases with GCS score < 11 (*P* < .001) and with intraventricular hemorrhage (*P* < .001) in patients with poor prognosis were 75%, 43.8%, and 78.1% respectively, higher than patients with good prognosis. The maximum diameter of aneurysms in patients with poor prognosis was 6.24 ± 2.89 mm, longer than patients with good prognosis (4.69 ± 2.60 mm), *P* = .007 (Table 1).

On the preoperative day, there was missing data in 1 patient due to lack of testing. The neutrophil count in patients with poor prognosis was (12.83 ± 5.39) × 10^9^/L, higher than patients with good prognosis ([10.09 ± 4.07] × 10^9^/L), *P* = .011. The monocyte counts in patients with good prognosis and poor prognosis were (0.42 ± 0.36) × 10^9^/L and (0.54 ± 0.49) × 10^9^/L respectively, with significant difference, *P* = .021. The SIRI level in patients with poor prognosis was (6.64 ± 10.46) × 10^9^/L, higher than patients with good prognosis (4.14 ± 5.72) × 10^9^/L, *P* = .046. There were no significant differences in levels of lymphocytes (*P* = .316), platelets (*P* = .416), SII (*P* = .342), NLR (*P* = .635) and PLR (*P* = .503) between patients with good prognosis and poor prognosis (Table 2).

On the 1st postoperative day, the neutrophil count in patients with poor prognosis was (10.77 ± 5.21) × 10^9^/L, higher than patients with good prognosis (8.74 ± 3.65) × 10^9^/L, *P* = .005. There were no significant differences in counts of lymphocytes (*P* = .282), monocytes (*P* = .147), and platelets (*P* = .935) between patients with good prognosis and poor prognosis. The SIRI level in patients with poor prognosis was (10.49 ± 9.62) × 10^9^/L, higher than patients with good prognosis (6.40 ± 6.60) × 10^9^/L, *P* = .006. The SII level in patients with poor prognosis was (2311.72 ± 2273.49) × 10^9^/L, higher than patients with good prognosis (1782.43 ± 1473.77) × 10^9^/L, *P* = .019. The levels of NLR in patients with good prognosis and poor prognosis were 9.97 ± 6.46 and 13.10 ± 11.52 respectively, with significant difference, *P* = .009 (Table 2).

**Figure 2.**
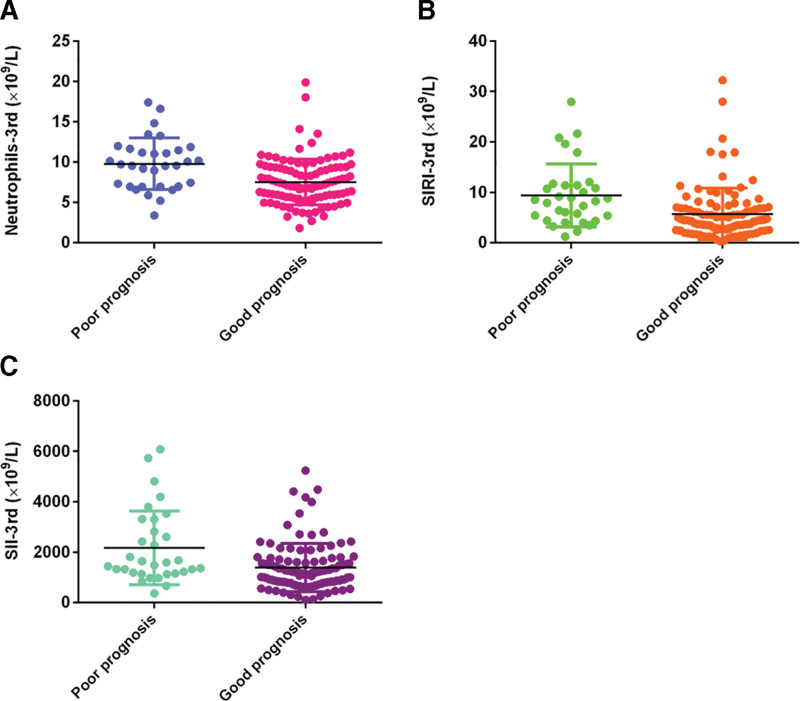
The differences of neutrophils (A), SIRI (B), and SII (C) on the 3rd postoperative day between patients with good prognosis and poor prognosis. SII = systemic immune inflammation index, SIRI = system inflammation response index.

On the 3rd postoperative day, the neutrophil count in patients with poor prognosis was (9.78 ± 3.21) × 10^9^/L, significantly higher than patients with good prognosis (7.54 ± 2.85) × 10^9^/L, *P* < .001 (Fig. 2A). There were no significant differences in counts of platelets (*P* = .459) and monocytes (*P* = .077) between patients with good and poor prognosis. The SIRI level in patients with poor prognosis was (8.39 ± 6.75) × 10^9^/L, significantly higher than patients with good prognosis (4.44 ± 4.13) × 10^9^/L, *P* < .001 (Fig. 2B). The SII level in patients with poor prognosis was (1535.94 ± 2017.53) × 10^9^/L, higher than patients with good prognosis (1183.47 ± 866.79) × 10^9^/L, *P* = .002 (Fig. 2C). The NLR levels in these patients were 10.23 ± 7.61 and 6.48 ± 5.14 respectively, with significant difference, *P* < .001 (Table 2).

### 3.3. Relationships of inflammatory indicators and prognosis of aSAH patients by multivariable analysis

After adjusting for baseline differences in Hunt-Hess grade, GCS score, combination with intraventricular hemorrhage and maximum diameter of aneurysm, the levels of neutrophils (odds ratio = 3.860, 95% CI: 1.399–10.651, *P* = .009), SIRI (odds ratio = 3.968, 95% CI: 1.432–10.992, *P* = .008) and SII (odds ratio = 3.313, 95% CI: 1.029–10.665, *P* = .045) on the 3rd postoperative day could predict poor prognosis. Two other factors that remained independent predictors of poor prognosis across each model included Hunt-Hess grade and Maximum diameter of aneurysm (all P < .05; Table 3).

**Table 3:** Multivariable logistic regression analysis of laboratory values as predictors of poor prognosis.

Models	Variable	B	S.E.	Wald	*P*	Adjusted odds ratio (95% CI)
1	Hunt-Hess Grade	2.196	0.526	17.398	<.001	8.987 (3.203–25.219)
	Maximum diameter of aneurysm	1.602	0.52	9.491	.002	4.963 (1.791–13.753)
	Combination with intraventricular hemorrhage	0.697	0.635	1.203	.273	2.007 (0.578–6.968)
	GCS Score	−0.193	0.695	0.077	.782	0.825 (0.211–3.221)
	Preoperative Neutrophils	0.532	0.631	0.709	.400	1.702 (0.494–5.868)
	Neutrophils on the 1st postoperative day	0.718	0.582	1.523	.217	2.051 (0.655–6.420)
	Neutrophils on the 3rd postoperative day	1.351	0.518	6.805	.009	3.860 (1.399–10.651)
2	Hunt-Hess Grade	1.928	0.65	8.793	.003	6.874 (1.922–24.579)
	Maximum diameter of aneurysm	1.617	0.535	9.151	.002	5.039 (1.767–14.368)
	Combination with intraventricular hemorrhage	1.073	0.582	3.396	.065	2.923 (0.934–9.146)
	GCS Score	-0.178	0.674	0.07	.792	0.837 (0.223–3.136)
	Monocytes on the 1st postoperative day	0.038	0.618	0.004	.952	1.038 (0.309–3.490)
	Monocytes on the 3rd postoperative day	0.405	0.557	0.528	.467	1.499 (0.503–4.467)
3	Hunt-Hess Grade	2.21	0.53	17.373	<.001	9.119 (3.225–25.782)
	Maximum diameter of aneurysm	1.64	0.524	9.802	.002	5.155 (1.846–14.390)
	Combination with intraventricular hemorrhage	0.807	0.595	1.839	.175	2.240 (0.698–7.187)
	GCS Score	0.094	0.706	0.018	.894	1.099 (0.275–4.385)
	Preoperative SIRI	0.038	0.622	0.004	.951	1.039 (0.307–3.518)
	SIRI on the 1st postoperative day	0.232	0.647	0.129	.720	1.261 (0.355–4.484)
	SIRI on the 3rd postoperative day	1.378	0.52	7.026	.008	3.968 (1.432–10.992)
4	Hunt-Hess Grade	2.356	0.521	20.475	<.001	10.55 (3.802–29.273)
	Maximum diameter of aneurysm	1.587	0.512	9.59	.002	4.887 (1.790–13.339)
	Combination with intraventricular hemorrhage	0.915	0.594	2.375	.123	2.498 (0.780–8.000)
	GCS Score	−0.018	0.678	0.001	.979	0.982 (0.260–3.710)
	SII on the 1st postoperative day	0.504	0.558	0.817	.366	1.655 (0.555–4.940)
	SII on the 3rd postoperative day	1.198	0.596	4.033	.045	3.313 (1.029–10.665)

**Figure 3.**
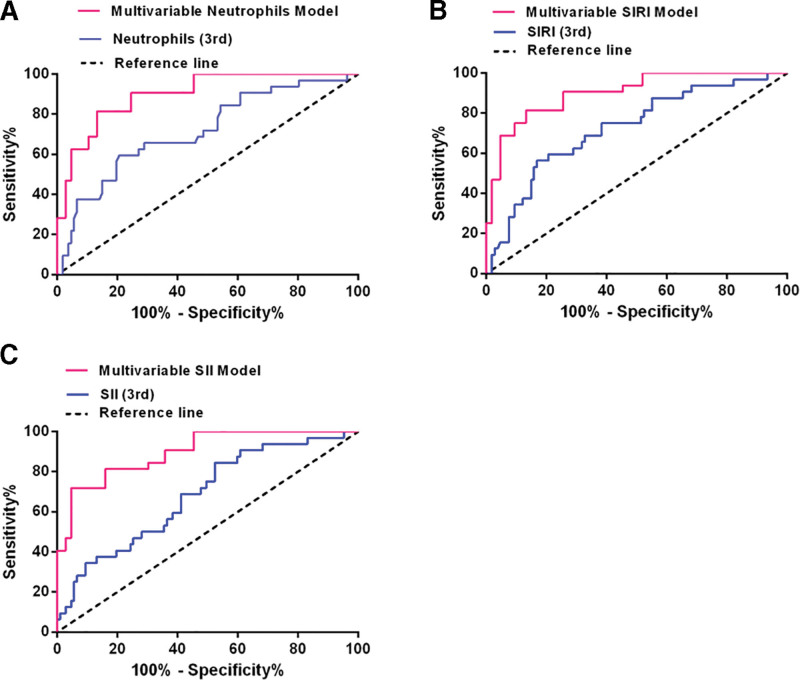
The ROC curves of neutrophils, SIRI, SII on the 3rd postoperative day and ROC curves of the multivariable models. The AUC of neutrophils was 0.715 (95% CI: 0.612–0.818, *P* < .001), and the AUC of multivariable neutrophils model was 0.867 (95% CI: 0.802–0.933, *P* < .001) (A). The AUC of SIRI was 0.725 (95% CI: 0.625–0.824, *P* < .001), and the AUC of multivariable SIRI model was 0.860 (95% CI: 0.782–0.938, *P* < .001) (B). The AUC of SII was 0.683 (95% CI: 0.580–0.785, *P* < .001), and the AUC of multivariable SII model was 0.855 (95% CI: 0.783–0.927, *P* < .001 (C). AUC = area under the curve, ROC = receiver operating characteristic curve, SII = systemic immune inflammation index, SIRI = system inflammation response index.

ROC analysis was performed to determine cutoff values to distinguish between aSAH patients with good prognosis and poor prognosis. The cutoff value of neutrophil was 9.48 × 10^9^/L. The area under the curve (AUC) of neutrophil on the 3rd postoperative day was 0.715 (95% CI: 0.612–0.818, *P* < .001). The sensitivity was 59.4% and the specificity was 79.4%. The Youden’s index was 0.388. We compared the ROC curves for neutrophils and the multivariable model including neutrophils (AUC = 0.867, 95% CI: 0.802–0.933, sensitivity 81.3%, specificity 75.5%, Youden’s index = 0.567, *P* < .001) (Fig. 3A).

The cutoff value of SIRI was 7.88 × 10^9^/L. The AUC of SIRI on the 3rd postoperative day was 0.725 (95% CI: 0.625–0.824, *P* < .001). The sensitivity was 56.3% and the specificity was 83.2%. The Youden’s index was 0.395. We compared the ROC curves for SIRI index and the multivariable model including SIRI (AUC = 0.860, 95% CI: 0.782–0.938, sensitivity 75.0%, specificity 86.8%, Youden’s index = 0.618, *P* < .001) (Fig. 3B).

The cutoff value of SII was 1118.93 × 10^9^/L. The AUC of SII on the 3rd postoperative day was 0.683 (95% CI: 0.580–0.785, *P* = .002). The sensitivity was 84.4% and the specificity was 47.7%. The Youden’s index was 0.321. We compared the ROC curves for SII index and the multivariable model including SII (AUC = 0.855, 95% CI: 0.783–0.927, sensitivity 71.9%, specificity 84.0%, Youden’s index = 0.558, *P* < .001) (Fig. 3C).

After covariate adjustment, the multivariable models including neutrophils, SIRI and SII all demonstrated significant improvement in distinguishing between patients with poor prognosis and good prognosis. The optimal predicted model was the SIRI multivariable model (Youden’s index = 0.618, sensitivity 75.0%, specificity 86.8%). The optimal cutoff value for SIRI level was 7.88 × 10^9^/L, whereby those with SIRI above this level would be likely to develop poor prognosis.

## 4. Discussion

Aneurysmal subarachnoid hemorrhage is a highly complex and fatal disease. Although improvement has been made in the treatment of aSAH in recent years, the prognosis of some patients is still poor.^[11]^ In recent years, there were some studies confirming the important role of neuroinflammation in the progression of stroke^[12–14]^ and exploring the relationships between inflammatory indicators and prognosis of patients. Counts of White blood cells and neutrophils were shown to be associated with the GCS score, the hemorrhage volume and the 90-day prognosis of cerebral hemorrhage patients.^[15]^ The immune response after cerebral hemorrhage could lead to a decrease in peripheral lymphocytes and an increase in cerebral lymphocytes, enhancing the inflammatory response and brain injury.^[16,17]^ We explored the relationships between inflammatory indicators including NLR, PLR, SII, and SIRI with the prognosis of aSAH patients in this study. The inflammatory response varied during different times of hospitalization after occurrence, so we investigated the inflammatory indicators on the preoperative day, the 1st and 3rd postoperative days.

NLR was the ratio of neutrophil to lymphocyte and confirmed to be associated with the severity or prognosis of most diseases.^[18,19]^ In recent years, the relationship between NLR and prognosis of stroke patients has been confirmed. In 1 study including 181 patients with cerebral hemorrhage, the mortality of cases with higher NLR (≥7.35) was 37.8% (28/74), while lower NLR (≤7.35) was 6.5% (7/107). Compared with lower NLR, cases with higher NLR also had higher incidence of intraventricular hemorrhage (29.7% vs 16.8%), higher volume of cerebral hemorrhage (23.9 vs 6.0 cm^3^) and lower GCS score (9.4 ± 4.5 vs 12.9 ± 3.2).^[20]^ In patients with aneurysmal subarachnoid hemorrhage, higher NLR was associated with poor prognosis. In the study by Giede Jeppe A et al,^[21]^ patients with higher mRS score (3–6) had significantly higher NLR (8.3 vs 5.8) when compared with lower mRS score (0–2). The NLR level at admission remained an important predictor of poor prognosis in SAH patients. Our study explored the relationship of NLR level on the preoperative, the 1st and 3rd postoperative days with the prognosis of aSAH patients. The result of univariable analysis showed that NLR level on the 1st and 3rd postoperative days in patients with poor prognosis was higher than patients with good prognosis. However, the results of multivariable logistic regression analysis did not confirm the association.

PLR was the ratio of platelet to lymphocyte, the importance of which varied in different tumor patients. In pancreatic cancer patients receiving neoadjuvant chemotherapy, there was a weak relationship between higher PLR and the increase of residual tumors.^[22]^ Among patients with metastatic or advanced gastric cancer who received PD-1 inhibitor treatment, patients with higher PLR before treatment had a lower disease control rate and objective response rate.^[23]^ Moreover, higher PLR was also a risk factor for poor efficacy of neoadjuvant chemotherapy in triple negative breast cancer.^[24]^ Higher PLR was also associated with poor vascular recanalization rate and poor prognosis at 3 months in patients with ischemic stroke.^[25]^ PLR was positively correlated with the percentage of carotid stenosis and was confirmed as an independent factor associated with stroke.^[26]^ There were few studies about the relationship between PLR and prognosis of aSAH patients.^[27]^ In our study, no relationship between PLR and prognosis was found in both univariable and multivariable analyses.

There were some studies on the relationships between SIRI and the prognosis of tumor patients. The overall survival of hepatocellular carcinoma patients with SIRI < 1.05 × 10^9^/L was longer than patients with SIRI ≥ 1.05 × 10^9^/L. The Cox multivariable regression analysis showed that SIRI level was associated with the overall survival and was superior to AFP level or Child-Pugh score in predicting the overall survival.^[28]^ Among breast cancer patients receiving chemotherapy, cases with SIRI ≥ 1.8 × 10^9^/L had shorter time to progression and shorter overall survival than patients with SIRI < 1.8 × 10^9^/L. Multivariable analysis confirmed that SIRI was an independent prognostic factor for TTP and overall survival.^[29]^ In a 20-Year follow-up cohort study of 42,875 US adults, cases with SIRI > 1.43 × 10^9^/L had a higher risk of all-cause death and cardiovascular death than cases with SIRI < 0.68 × 10^9^/L.^[30]^ Higher level of SIRI was also associated with higher stroke risk.^[31]^ In our study, univariable and multivariable analyses all confirmed higher SIRI level on the 3rd postoperative day was associated with poor prognosis in aSAH patients. Especially, the multivariable model including SIRI demonstrated significant improvement in distinguishing between patients with poor prognosis and good prognosis (Youden’s index = 0.618, sensitivity 75.0%, specificity 86.8%). The optimal cutoff value for SIRI level was 7.88 × 10^9^/L, whereby those with SIRI index above this level would be likely to develop poor prognosis.

In patients with coronary artery disease, higher SII was associated with higher risk of cardiac death, nonfatal myocardial infarct and nonfatal stroke.^[32]^ In patients with endometrial cancer, SII was an independent factor associated with overall survival.^[33]^ Among patients with gastric cancer undergoing radical surgery, the 5-year overall survival in patients with lower SII was significantly longer than patients with higher SII (92% vs 80%), especially in elderly (91% vs 73%) and stage-II patients (86% vs 67%). The cutoff value of SII was 508.3 × 10^9^/L.^[34]^ SII was also a predictor of disease severity. In acute pancreatitis, patients with SII ≥ 2207.53 × 10^9^/L had a higher proportion of developing SAP. SII was more sensitive and specific in predicting the severity of acute pancreatitis than NLR and PLR.^[35]^ Among patients receiving percutaneous nephrolithotomy, the AUC (0.782) of SII for predicting systemic inflammatory response syndrome was higher than NLR (0.671) and PLR (0.617).^[36]^ Among stroke patients, higher SII was also associated with poor outcome and high mortality.^[37]^ There was few study exploring the relationship of SII with the prognosis in aSAH patients. In our study, univariable and multivariable analyses all confirmed higher SII level on the 3rd postoperative day was associated with poor prognosis in aSAH patients. Especially, the multivariable model including SII demonstrated improvement in distinguishing between patients with poor prognosis and good prognosis. The cutoff value of SII for predicting poor prognosis was 1118.93 × 10^9^/L in our study, which was higher than in study by He K (508.3 × 10^9^/L)^[34]^ and study by Wang C (748.51 × 10^9^/L).^[38]^

Our study also had several limitations. First, the study mainly investigated the relationships of blood cell counts and related indicators with the prognosis of aSAH patients, but did not investigate other inflammatory indicators including C-reactive protein, interleukin-6 and other inflammatory factors. Second, the sample size was not big enough, and we will expand the sample size for the next step. Third, we did not study the dynamic changes of these inflammatory indicators.

## 5. Conclusion

SIRI and SII on the 3rd postoperative day were possibly superior predictors for the prognosis in aSAH patients compared to other inflammatory indicators. We should pay attention to the SIRI and SII levels during different hospitalization periods, especially in the short postoperative period. Moreover, the cutoff values for predicting prognosis need to be validated in larger-sample studies.

## Acknowledgments

We are thankful to all the medical staff and statistician in the Neurointensive Care Unit, The First Affiliated Hospital of Yangtze University.

## Author contributions

**Conceptualization:** Xian Wang, Li Deng.

**Data curation:** Xian Wang, Wei Tian, Yongfeng Zhao.

**Formal analysis:** Wei Tian, Yongfeng Zhao.

**Methodology:** Yongfeng Zhao, Yong Yang.

**Project administration:** Li Deng.

**Writing – original draft:** Xian Wang, Yongfeng Zhao.

**Writing – review & editing:** Li Deng.
